# Hypertriglyceridemic Waist Phenotype: Effect of Birthweight and Weight Gain in Childhood at 23 Years Old

**DOI:** 10.1371/journal.pone.0134121

**Published:** 2015-08-26

**Authors:** Ricardo Lanzetta Haack, Bernardo Lessa Horta, Denise Petrucci Gigante, Fernando C. Barros, Isabel Oliveira, Vera M. F. Silveira

**Affiliations:** Post-Graduate Program in Epidemiology, Federal University of Pelotas, Pelotas-RS, Brazil; School of Public Health, Zhejiang University, CHINA

## Abstract

**Objective:**

To evaluate the association of birthweight and weight gain during different periods in childhood with the prevalence of hypertriglyceridemic waist phenotype (HWP).

**Methods:**

In 1982, all hospitals births in Pelotas, South Brazil, were identified, and the 5914 liveborn were examined and their mothers interviewed. This population has been followed for several times. In 2004–05, we tried to follow the whole cohort and the subjects were interviewed, examined, and a blood sample was collected. HWP was defined as a triglycerides ≥ 2 mmol/L and a waist circumference ≥ 90 cm for men, and triglycerides ≥ 1.5 mmol/L and waist circumference ≥ 85 cm for woman. Poisson regression with robust adjustment of the variance was used to obtain adjusted estimates of the prevalence ratio.

**Results:**

Subjects whose weight-for-age z-score at mean age of 42 months was one or more standard deviation above the mean, according to gender and age, were 8.77 (95% confidence interval: 2.60; 29.64) times more likely of presenting the HWP than those subjects whose weight-for-age z-score at 42 months was more than one standard deviation below the mean. Among those subjects whose birthweight was adequate-for-gestational age (AGA), conditional weight at 20 months was positively associated to the risk of HWP [relative risk: 1.59 (95%: confidence interval: 1.32; 1.92)], whereas for small for gestational age (SGA) subjects conditional weight was not associated with HWP [relative risk: 1.05 (95% confidence interval: 0.77; 1.43)], p-value for interaction 0.08.

**Conclusion:**

Early weight gain among SGA infants, did not increase the risk of HWP in early adulthood, whereas among those who were AGA, early weight gain increased the risk of the having the phenotype in early adulthood.

## Introduction

It has been suggested that the development of noncommunicable diseases may be programmed by exposures in early life.[1, 2] Intrauterine malnutrition would increase the risk of cardiovascular disease in adulthood[3], and blood pressure in adulthood is inversely related to birthweight.[4] On the other hand, other studies have reported that the development of chronic diseases is programmed by postnatal not by intrauterine growth.[5, 6]

Concerning weight gain in childhood, evidence on its long-term consequence is conflicting. Catch-up in early childhood has been associated with increased birthweight in the next generation[7] and achieved schooling.[8, 9] Meta-analysis by Owen et al [10] observed that body mass index (BMI) in early childhood was not related to the risk of coronary heart disease (CHD), whereas BMI in later childhood and early adulthood was associated with an increased risk of CHD.[10] Furthermore, rapid weight gain after 4 years, among individuals who were light at birth, was positively associated with systolic blood pressure in adulthood.[6] In the same token, Crowther et al reported that catch-up in the first year of life was not related to insulin and glucose levels, whereas weight gain after the first year would lead to higher insulin level.[11] Another study in Philippines observed that homeostasis model assessment of insulin resistance (HOMA-IR) at 22 years was not related to weight gain from 0 to 4 months, but weight gain from 0 to 2 years was positively related to HOMA-IR among males and this association was mediated by body mass index and waist circumference in adulthood.[12] In order to help to solve the catch-up dilemma, studies should assess the consequence of rapid growth in different periods in childhood, because evidences suggest that timing of growth may have different long-term consequences.

The hypertriglyceridemic waist phenotype (HWP) has been associated with the presence of cardiometabolic risk profile (increased levels of insulin, Apolipoprotein B, C-reactive protein and small dense LDL cholesterol) and an increased risk of coronary artery disease. Arsenault et al observed that even after controlling for cardiovascular risk factors (metabolic and behavior), subjects with HWP had a higher hazard of coronary artery disease [1.28 (95%confidence interval: 1.07; 1.54) for males, and 1.67(95% confidence interval: 1.35; 2.06) for females)].[13] Furthermore, it has been reported that impaired fasting glucose is not related with coronary artery disease, among subjects that does not present the HWP.[14] These findings shows the relevance of the phenotype as a marker of cardiovascular risk. To our knowledge, the programming of the phenotype by early growth has not been previously evaluated. By assessing the effect of weight gain during different periods in childhood on the phenotype, this study may help to solve the catch-up dilemma.

This study was aimed at assessing the effect of birthweight and growth in different periods on the development of the hypertriglyceridemic waist phenotype.

## Material and Methods

In 1982, all hospitals births in Pelotas, South Brazil (current population 320.000), were identified, and the 5914 liveborn whose family lived in the urban area of the city were examined and their mothers interviewed. This population has been followed for several times. In 1984 (mean age 20 months) and 1986 (mean age 42 months), all households in the city were visited in search of children belonging to the cohort; 87 and 84% of the original cohort were located, respectively. From October 2004 to August 2005 (mean age 23 years), all households located in urban area of the city were visited in search of cohort members. For those who had not been located and were not known to have died, we used the last known address and existing databases (including universities, secondary schools and telephone directories) for another attempt. The subjects answered a questionnaire on sociodemographic, health and behavioral variables. At the end of the interview, the subjects were invited to visit the research laboratory to give a blood sample. Another home visit was made, with the aim of obtaining blood samples from the interviewees who did not go to the laboratory. Further details on the methodology of the study are available elsewhere.[15]

Birthweight was assessed by the maternity hospital staff using calibrated scales; low birthweight was defined as <2500 g. Gestational age was calculated according to the recalled date of the mother’s last menstrual period, and preterm birth was defined as gestational age <37 weeks. Those children whose birthweight was below the 10th centile for gestational age and sex, according to the reference developed by Williams et al,[16]were classified as small-for-gestational age (SGA).

In 1984 and 1986, subjects were weighed using a portable scale with an accuracy of 100 g and the length (1984) and height (1986) were measured using a portable stadiometer. Birthweight for gestational age z-scores were calculated using the Williams’s reference.[16] In the follow-up visits, z-scores according to weight-for-age and sex were estimated, using the World Health Organization (WHO) standard.[17] Waist circumference was measured at the narrowest girth of the trunk or halfway between the costal margin and iliac crest, using a flexible 160cm (precision: 1mm) fiberglass measuring tape. Triglyceride was assessed with a colorimetric enzymatic method.

Hypertriglyceridemic waist phenotype was defined as triglycerides ≥ 2 mmol/L and waist circumference ≥ 90 cm for men[18], whereas for women triglycerides ≥ 1.5 mmol/L and waist circumference ≥ 85 cm were used as cut-off.[19]

Poisson regression with robust adjustment of the variance was used to obtain adjusted estimates of the prevalence ratio.[20] The following variables were considered as possible confounders: family income; household assets index (obtained through factor analysis and based on the ownership of household goods); parental schooling at birth; maternal smoking during pregnancy; maternal age; and maternal prepregnancy body mass index.

Conditional regression was used to take into account the correlation between subsequent weight measures and regression to the mean.[21] Weight-for-age z-score at 20 months was predicted from birthweight for gestational age z-score, and the difference (residual) between the observed and predicted weight-for-age z-score at 20 months was estimated. This residual was included in the analysis that assessed the effect of weight gain in the first 20 months. Weight-for-age z-score at 42 months was predicted using a similar approach, and the regression included birthweight for gestational age z-score and weight-for-age z-score at 20 months.

The confidentiality of all information was ensured and written informed consent was obtained in all phases of the study, when participants were minors written consent was obtained from their parents or guardians. The Medical Ethics Committee of the University of Pelotas, affiliated with the Brazilian Medical Research Council, approved the research protocol.

## Results

In the 2004–5 follow-up visit, 4297 subjects were interviewed, representing a follow-up rate of 77.4% (added to the 282 known to have died), and a blood sample was collected from 3,914 individuals. Table 1 shows that among those subjects studied in 2004–5, the prevalence of low birthweight was 6.2% and 7.9% for male and female, respectively. In 1984, at a mean age of 20 months, 3.5% of the males and 2.7% of the females had a weight for age z-score <- 2 standard deviation. In early adulthood, triglyceride was higher and HDL cholesterol was lower among male. The prevalence of hypertriglyceridemic waist phenotype was 5.9% and 4.5% among males and females, respectively.

**Table 1:** Distribution of sample studied at 23 years of age, according to key characteristics.

Sample characteristics			Men	Women
**At birth (1982)**				
Birthweight in grams, mean (SD)			3279 (523)	3163 (503)
Low birthweight, n (%)			136 (6.2)	165 (7.9)
Preterm birth, n (%)			97 (5.5)	86 (5.2)
Small-for-gestational age, n (%)			268 (15.1)	229 (13.8)
**1984 follow-up visit**				
Weight for age z-scores, n (%)				
	<- 2		70 (3.5)	51 (2.7)
	- 2 to -1.01		247 (12.2)	187 (9.7)
	- 1 to 0.99		1309 (64.9)	1323 (68.7)
	≥ 1		392 (19.4)	364 (18.9)
Weight for age in z-scores, mean (SD)			0.06 (1.11)	0.13 (1.04)
**1986 follow-up visit**				
Weight for age z-scores, n (%)				
	<- 2		35 (2.0)	37 (2.3)
	- 2 to -1.01		225 (13.0)	218 (13.6)
	- 1 to 0.99		1199 (69.4)	1121 (69.7)
	≥ 1		269 (15.6)	232 (14.3)
Weight for age in z-scores, mean (SD)			0.01 (1.06)	-0.03 (1.02)
**2004/5 follow-up visit**				
Triglycerides in mg/dL, mean* (IQR)			97.3 (78)	85.9 (56)
HDL cholesterol in mg/dL, mean (SD)			51.6 (11.2)	59.4 (13.4)
Waist circumference in cm, mean (SD)			80.9 (10.1)	74.7 (10.5)
Hypertriglyceridemic waist phenotype, n(%)			113 (5.9)	80 (4.5)

* geometric mean

Table 2 shows that birthweight for gestational age z-score and weight-for-age z-score in childhood was positively related to the risk of having the HWP. Those subjects whose weight-for-age z-score at mean age of 42 months was one or more standard deviation above the mean were 8.77 (95% confidence interval: 2.60; 29.64) times more likely of presenting the hypertriglyceridemic waist phenotype than those whose weight-for-age z-score at 42 months was more than one standard deviation below the mean.

**Table 2:** Prevalence ratio of hypertriglyceridemic waist phenotype according to birthweight for gestational age and weight for age z-score at 20 and 42 months.

		Prevalence ratio of hypertriglyceridemic waist phenotype (95% confidence interval)		N
		Crude	Adjusted	
Birthweight for gestational age z-score		P = 0.09 **	P = 0.03 **	
	< -1	Reference (1)	Reference (1)	433
	-1 to 0.99	0.93 (0.63; 1.37)	1.07 (0.66; 1.74)	1302
	≥ 1	1.76 (1.08; 2.87)	1.92 (1.05; 3.50)	1213
Weight for age z-scores at mean age of 20 months #		P = 0.002 *	P = 0.04 *	
	<- 1	Reference (1)	Reference (1)	474
	- 1 to 0.99	1.30 (0.78; 2.14)	1.71 (0.73; 4.01)	2260
	≥ 1	2.14 (1.25; 3.67)	2.39 (0.94; 6.03)	651
Weight for age z-scores at mean age of 42 months #		P < 0.001 *	P < 0.001 *	
	<- 1	Reference (1)	Reference (1)	515
	- 1 to 0.99	2.35 (1.24; 4.47)	4.11 (1.27; 13.20)	2320
	≥ 1	5.86 (3.03; 11.3)	8.77 (2.60; 29.64)	501

* test for linear trend

** test for heterogeneity adjusted for household assets, family income, parental schooling at birth, maternal smoking during pregnancy, maternal age, and maternal prepregnancy body mass index.

# also adjusted for birthweight according to the gestational age z-score.

**Fig 1:**
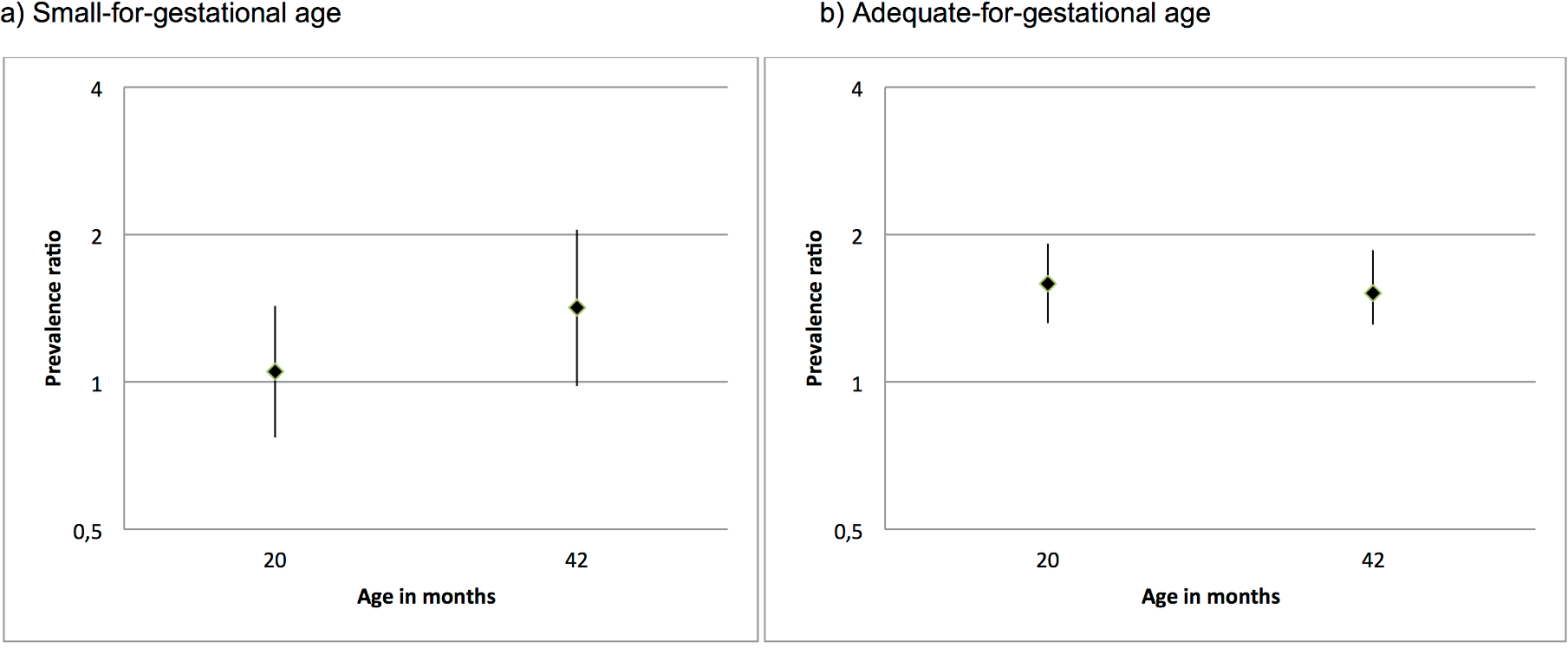
Adjusted * prevalence ratio of hypertriglyceridemic waist phenotype according to predicted weight at the mean ages of 20 and 42 months in SGA (a) and AGA (b) subjects. * adjusted for birthweight according to the gestational age z-score, household assets, family income, parental schooling at birth, maternal smoking during pregnancy, maternal age, and maternal prepregnancy body mass index.

Table 3 shows that early and late weight gain in childhood were related to a higher risk of having the HWP. On the other hand, Fig 1 shows that the effect of weight gain in early childhood was modified by intrauterine growth. Among those subjects whose birthweight was adequate-for-gestational age [22], conditional weight at 20 months was positively associated to the risk of having the HWP [relative risk: 1.59 (95%: confidence interval: 1.32; 1.92)], whereas conditional weight was not associated with the HWP [relative risk: 1.05 (95% confidence interval: 0.77; 1.43)] among SGA subjects; but the test for interaction was not significant (p-value = 0.08).

**Table 3:** Adjusted* conditional growth analyses of hypertriglyceridemic waist phenotype according to predicted weight at the mean ages of 20 and 42 months.

		Prevalence ratio of hypertriglyceridemic waist phenotype (95% confidence interval)	
		Crude	Adjusted
Weight at 20 months minus predicted weight (Z-scores) &	Coefficient (95% confidence interval)	1.50 (1.27; 1.76)	1.50 (1.27; 1.78)
	P-value	< 0.001	< 0.001
Weight at 42 months minus predicted weight (Z-scores) $	Coefficient (95% confidence interval)	1.51 (1.32; 1.72)	1.51 (1.32; 1.72)
	P-value	< 0.001	< 0.001

* adjusted for household assets, family income, parental schooling at birth, maternal smoking during pregnancy, maternal age, and maternal prepregnancy body mass index.

& Also adjusted for birthweight

$ Also adjusted for birthweight and weight residual at 20 months

**Fig 2:**
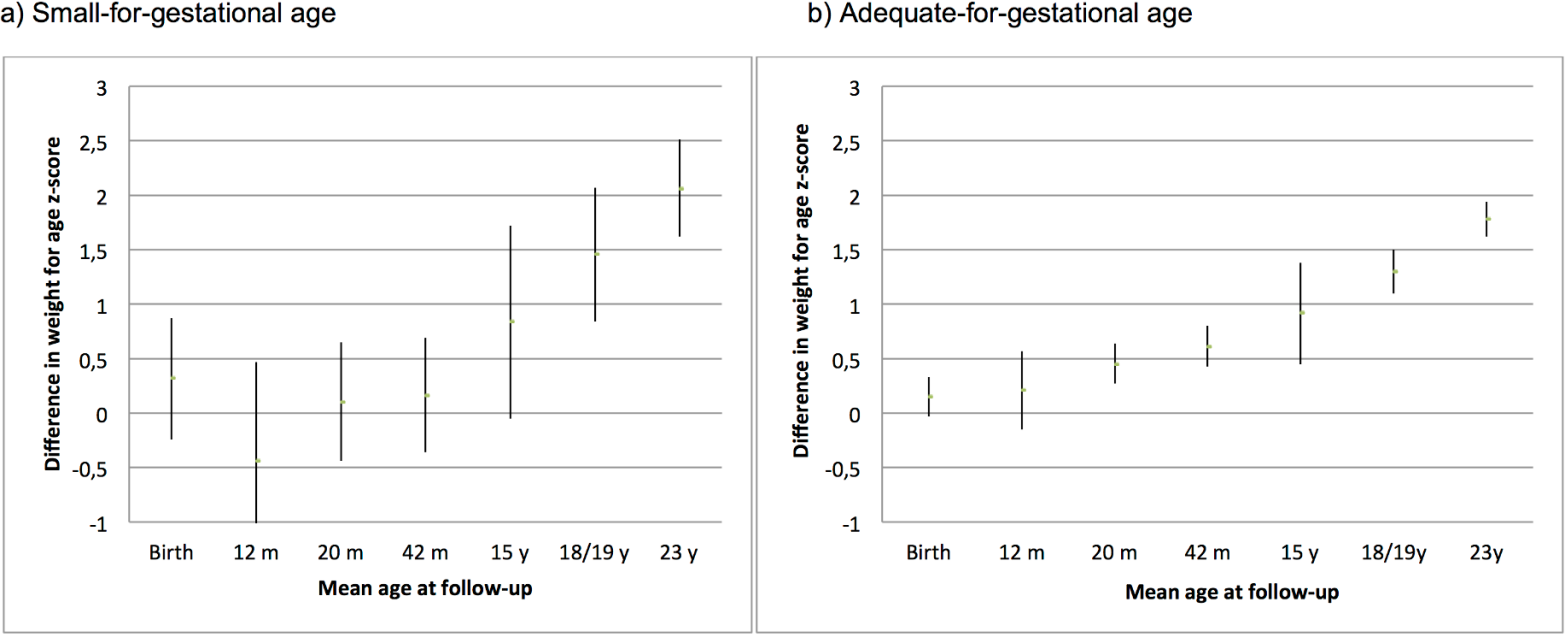
Mean sex specific adjusted* weight-for-age z-scores of subjects classified as having the hypertriglyceridemic waist phenotype, according to intrauterine growth. **The mean weight for age z-score in each strata (small and adequate-for-gestational age) was set to zero, with deviations from the mean expressed as standard deviations (z-scores).** * adjusted for birthweight according to the gestational age, household assets, family income, parental schooling at birth, maternal smoking during pregnancy, maternal age, and maternal prepregnancy body mass index.

Fig 2 shows the weight trajectory from birth to 23 years of age among those subjects who presented the hypertriglyceridemic waist phenotype. In this analysis, each measurement among SGA and adequate for gestational age (AGA) infants was standardized, with mean set at zero, and the deviations from the mean are shown in standard deviations. Therefore, the values presented in Figs 1 and 2 shows the difference in weight-for-age z-scores between those who presented and not the phenotype. A mean below zero indicated that weight-for-age was small among those with HWP. The results were very similar to that observed with the conditional growth model. Among AGA subjects, we observed a steady increase in the difference in weight between those with and without the hypertriglyceridemic waist phenotype, whereas for SGA subjects in the first 20 months this difference did not change, with the mean difference near to zero, but after that a steady increase in the difference was also observed. Suggesting that the early weight gain among AGA infants increased the risk of the having the phenotype in early adulthood. On the other hand, early weight gain among SGA infants, may not increase the risk of HWP in early adulthood, but these findings need to be replicated in a large sample.

## Discussion

In this cohort that has been followed since birth, in a southern Brazilian city, we observed that birthweight was not related to hypertriglyceridemic waist phenotype in early adulthood. On the other hand, weight-for-age z-score in childhood was positively associated with the risk of presenting the phenotype. With respect to weight gain in childhood, among those subjects who were born small-for-gestational age, weight gain in the first 20 months was not related to the risk of having the phenotype, whereas weight gain from 20 to 42 months increased the risk. On the other hand, among adequate-for-gestational subjects, early and late weight gain in childhood increased the risk of having the phenotype.

With respect to the validity of the evidence, the use of standardized methods in the anthropometric evaluation in childhood minimized misclassification error. Moreover, confounders were prospectively evaluated, using standardized questionnaires and trained interviewers, reducing the likelihood of residual confounding. Follow-up rates were not related to birthweight and maternal skin color. On the other hand, subjects of families whose income was either at the lower or upper end of the distribution and those whose mother had 12 or more years of schooling were less likely to the followed in adulthood.[23]

Weight gain in childhood and birthweight were not associated with losses in the follow-up visit in 2004–5. Therefore, the study is less susceptible to selection bias. Because triglycerides were measured from non-fasting blood, this study would be susceptible to misclassification, but evidence suggests that triglycerides measured from non-fasting blood predict risk of cardiovascular disease better than fasting levels.[22, 24–26] Therefore, the use of non-fasting triglycerides in the analysis of long-term consequences of weight gain on childhood on risk of HWP is acceptable. As previously mentioned, the hypertriglyceridemic waist phenotype is related to the presence of cardiometabolic risk factors and coronary artery disease.[13] Therefore, it should be considered as a marker of higher cardiovascular risk.

Few studies have evaluated the long-term consequences of rapid weight gain in childhood on triglycerides and waist circumference. In our cohort, we have already evaluated the effect of early weight gain on blood lipids among18 years old males, and weight gain from birth to 20 months of age was not associated with blood lipids. However, rapid weight gain from 20 to 42 months of age was positively associated to very low-density lipoprotein (VLDL), low-density lipoprotein cholesterol (LDL) / high-density lipoprotein cholesterol (HDL) ratio, and triglycerides.[27] Other studies have also reported that weight gain in later childhood is related to increased blood lipids level. [28–30]

There is evidence that genotypes are associated with obesity in adulthood and growth in childhood,[31] [32] and also can affect the development of HWP. On the other hand, the absence of an effect of early growth among those who were born small-for-gestational age suggests that the observed association is probably due to an environmental factor instead of genetic.

Weight gain in childhood has been used as synonymous of catch-up growth, but we should be careful before labeling every weight gain as catch-up. Tanner[33] defined catch-up growth as the accelerated growth that occurs as soon as an insult is removed and growth failure ends. In the present study, we were able to assess the effect of catch-up growth, by stratifying the analysis according to intrauterine growth; and observed that early weight gain among small-for-gestational age infants, i.e. catch-up growth, did not increase the risk of presenting the hypertriglyceridemic waist phenotype in early adulthood, whereas among those whose birthweight was adequate for gestational age, early weight gain increased the risk of the having the phenotype. Suggesting, therefore, that catch-up growth in early childhood is not related to the programming of cardiovascular diseases. On the other hand, the small number of SGA subjects included in the analysis reduced the statistical power of the study to assess the interaction between early growth and intrauterine growth. Therefore, these findings need to be replicated in another studies.

Early rapid weight gain has short-term benefits, reducing mortality and morbidity.[34] Furthermore, evidence suggests rapid weight gain in early childhood is positively associated with human capital[8, 9] and is not associated to the presence of metabolic cardiovascular risk factors. [6, 27, 35] Therefore, weight gain should be stimulated in early childhood, mainly among those infants who were underweight.

## Supporting Information

S1 Database Conditional Grow. (ZIP)

S2 Database Nutritional Status. (ZIP)
